# Faut-il préférer une technique chirurgicale dans le traitement des ruptures du tendon d'Achille?

**DOI:** 10.11604/pamj.2015.20.285.5700

**Published:** 2015-03-24

**Authors:** Redouane Hani, Mohammed Kharmaz, Mohammed Saleh Berrada

**Affiliations:** 1 Service de Chirurgie Traumatologique et Orthopédique, CHU Ibn Sina, Rabat, Maroc

**Keywords:** Rupture tendon d´Achille, traitement chirurgical, ténorraphie percutanée, Bosworth, Chigot, complications, Achilles tendon rupture, surgical treatment, Percutaneous tenorrhaphy, Bosworth, Chigot, complications

## Abstract

La rupture du tendon d'Achille est de plus en plus fréquente dans le monde et dans notre pays en raison du développement considérable des activités sportives, de l'accroissement de leur intensité et de l'absence de moyens de prévention. Notre travail porte sur une étude concernant 58 cas de rupture du tendon d'Achille, avec un recul moyen compris entre 5 mois et 80 mois. L’âge moyen était de 36 ans, tous les patients inclus ont tous bénéficier d'un traitement chirurgical. Le but de notre étude étant de souligner la supériorité d'une technique chirurgicale par rapport à une autre dans la prise en charge, ainsi qu'une mise au point sur les différents aspects épidémiologiques, cliniques, thérapeutiques et post-thérapeutiques de cette lésion.

## Introduction

La rupture du tendon d'Achille se définie comme une solution de continuité intéressant une partie ou la totalité de sa largeur. Le tendon d'Achille est généralement considéré comme le tendon le plus épais et le plus fort de l'organisme, sa rupture est connue depuis l'Antiquité, mais sa description princeps revient à Ambroise Paré qui, en 1575, traita le roi Charles IX pour cette lésion par repos allongé.

Son incidence a longtemps été considérée comme rare; le développement marqué des activités sportives et de loisir l'a vue considérablement augmenter actuellement dans le monde et dans notre pays où il s'agissait autrefois de section par objet tranchant, selon les enquêtes épidémiologiques, avec une prédominance masculine entre 30 et 50 ans. Elles sont le plus souvent la conséquence des lésions dégénératives dues aux microtraumatismes et aux surmenages tendineux liés presque toujours au sport et à l'hyperactivité. Si le diagnostic de la lésion est aisé, son traitement prête aujourd'hui encore à controverse.

De nouvelles études parues dans la littérature spécialisée tendent, toutefois, à démontrer que le traitement chirurgical permet d'atteindre de meilleurs résultats, principalement chez un patient jeune et sportif. Le traitement chirurgical en phase aigüe n'est pas sans risque de complications, surtout dans les techniques traditionnelles. Dans le but de minimiser ces complications, le traitement de ces lésions a connu des progrès considérables grâce à l'avènement de méthodes thérapeutiques nouvelles tel que: le traitement fonctionnel, chirurgie percutanée et chirurgie mini invasive qui sont moins invasives. Au Maroc, on tend souvent vers un traitement chirurgical. Mais y a-t-il une différence en matière de résultats fonctionnels en fonction de la technique chirurgicale utilisée?

## Méthodes

Il s'agit d'une étude rétrospective concernant 58 observations répertoriées au Service de traumatologie et d'orthopédie de l'Hôpital Avicenne de Rabat sur une durée de 7 ans entre Janvier 2007 et Décembre 2013, avec un recul entre un minimum de 5 mois et un maximum de 6 ans et 7 mois. Les patients étaient inclus dans l’étude selon les critères suivants: rupture du tendon d'Achille; récente ou ancienne; partielle ou totale; sous cutanée ou suite à une plaie.

## Résultats

**Figure 1:**
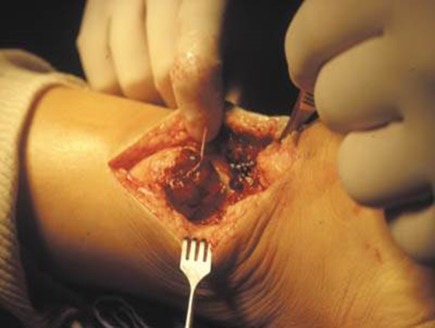
Rupture fraiche de tendon d'Achille

**Figure 2:**
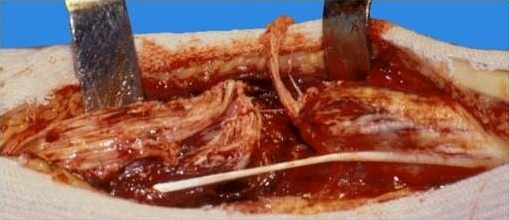
Rupture fraiche de tendon d'Achille tendant plantaire grêle intact

L’âge de nos patients varie entre 16 ans et 58 ans. Le maximum des cas se situait entre 30 et 50 ans, selon une fréquence de 8,2 cas par an. La majorité des cas dans notre série était de sexe masculin (51 Homme et 7 femmes). Douze de nos patients ont présenté des antécédents en rapports avec la rupture du tendon d'Achille. Ainsi, huit patients ont rapporté une notion de prise de corticoïdes à long terme, deux autres avaient un antécédent de rupture ancienne, et deux autres patients était suivi pour hypercholestérolémie. Six de nos patients ont présenté une rupture ouverte du tendon d'Achille contre 52 cas de rupture sous cutanée, sans prédominance d'un côté par rapport à l'autre, vu que le côté droit a été atteint chez 32 patients, alors que le côté gauche chez 26 patients Figure 1 et Figure 2. Les circonstances qui ont occasionné les ruptures du tendon d'Achille dans notre série ont été dominées par les accidents de sport (43 cas soit 74.2%). Venaient ensuite les accidents domestiques et du travail qui ont été observés dans sept cas soit (12,1%). Les traumatismes ont été incriminés dans huit cas (13,7%).

Le délai du diagnostic était plus de huit jours dans 11 cas et moins de huit jours dans 47. Toutes les ruptures étaient évidentes à l'examen clinique qui était souvent gêné en raison de la douleur. Ainsi, en position debout, la boiterie est constante et l'appui monopodal est impossible. En décubitus ventral, le signe de Brunet-Guedj est présent et la manoeuvre de Thompson est positive chez tous les patients. Les examens paracliniques n’étaient jamais indispensables au diagnostic. Cependant, tous les patients ont bénéficié systématiquement d'une radiographie standard de la cheville qui a montré une perte de l’équinisme physiologique sans fractures osseuses associées. L’échographie a été réalisée pour 21 cas (36,8%) confirmant le diagnostic. L'IRM n'a été réalisée chez aucun patient.

Le traitement était en fonction de l’âge du patient, du sport pratiqué, et de la demande du patient. Tous les patients de notre série ont bénéficié d'un traitement chirurgical à ciel ouvert par suture, laçage ou par plastie suivi d'une immobilisation. Une rachianesthésie a été pratiquée dans 88,5% des cas. L'installation était chez tous nos patients en décubitus ventral avec garrot pneumatique à la racine de la cuisse.

La voie d'abord était para- achilléenne interne, 2 à 3 cm en dedans du milieu de la face postérieure du tendon, et prolongée de 5 à 6 cm vers le haut dans les cas où on a utilisé la technique de Bosworth. L'exploration chirurgicale trouve une rupture totale dans la grande majorité des cas (56 cas) en plein corps tendineux notamment au niveau du 1/3 moyen. La technique chirurgicale la plus utilisée dans notre série était le Bosworth dans 48.2% des cas, suivie de la technique de Chigot dans 31.1% des cas, et les sutures termino-terminales dans 20.7% des cas. Une attelle plâtrée en équin physiologique a été réalisée au bloc opératoire pour permettre de surveiller l’état cutané pendant les 48 premières heures, relayée par une botte plâtrée en équin puis, au bout de trois semaines, par une botte plâtrée à 90° pour trois semaines supplémentaires, puis par des plâtres itératifs permettant de récupérer progressivement une position à 90° de la tibio-tarsienne. L'appui a été repris progressivement et la rééducation a été suivie par tous nos patients.

Une infection superficielle a été relevée et a été traitée par une bi-antibiothérapie synergique et bactéricide, après prélèvement bactériologique et antibiogramme, associée aux soins locaux. L’état cutané a bien évolué par la suite. Aucun cas de rupture itérative n'a été rapporté, ni de douleurs résiduelles. Enfin, aucune atteinte du nerf sural ni complication thrombo-embolique n'ont été signalées.

## Discussion

Durant ces deux dernières décennies, de multiples auteurs ont rapporté une augmentation de l'incidence des ruptures du tendon calcanéen [1]. L'une des explications retenues devant l'augmentation du nombre de ruptures ces vingt dernières années est le gain de popularité des sports de loisirs. L'incidence annuelle des ruptures du tendon d'Achilles est passée par exemple de 18,2/100000 habitants en 1984 pour 37,3/100000 habitants en 1996 au Danemark [2].

**Figure 3:**
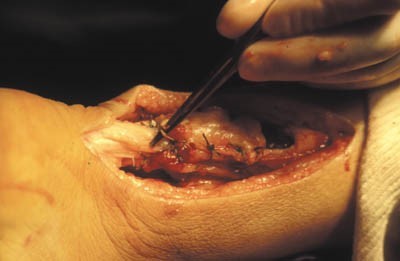
Tenorraphie avec plastie selon Bosworth

**Figure 4:**
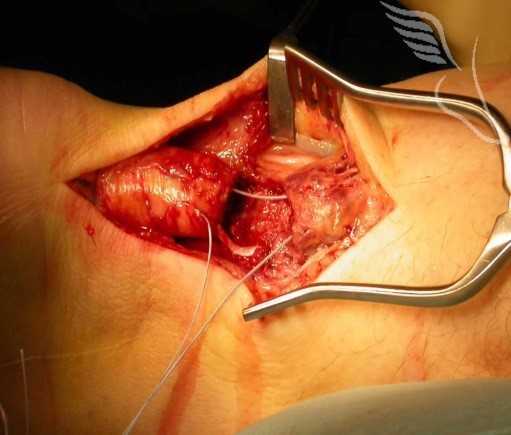
Suture termino- terminale de tendon d'Achille

Notre série comporte 58 cas de rupture du tendon d'Achille, exploités sur une période de sept ans. Ce chiffre se rapproche des données de la littérature, ceci peut être expliqué par le développement considérable des activités de sport dans notre pays. L’âge de nos malades varie entre 16 et 58 ans avec un âge moyen de 36 ans, qui s'avère inférieur par rapport à certaines séries [3, 4]. Ceci peut être expliqué par la présence d'une population jeune au Maroc. La prédominance masculine est admise dans toutes les séries. Elle est de 94,7% dans notre série. La majorité des patients de notre série ont eu une rupture lors d'une activité sportive ainsi la cause la plus fréquente des ruptures du tendon d'Achille est représentée par les accidents de sport ce qui a été rapporté dans toutes les séries de la littérature. Les prises médicamenteuses et les tendinites sont incriminées dans la genèse de la rupture du tendon d'Achille, ce qui justifie la nécessité de la prévention et de la prise en charge précoce des tendinopathies. L'incidence des prises médicamenteuses et des tendinites reste faible dans la majorité des séries de la littérature. Dans notre série, huit patients avaient un antécédent de prise de corticoïdes. Le diagnostic est facile et ne devrait pas être méconnu en urgence grâce à un interrogatoire simple et un examen clinique rigoureux. Dans notre série l'interrogatoire et l'examen clinique étaient faciles et suffisants pour poser le diagnostic, ce qui correspond aux données de la littérature où les examens complémentaires ne sont faits que pour éliminer d'autres lésions (radiographie standard) ou à titre complémentaire (Echographie et IRM) [5]. En comparant nos résultats avec ceux des autres séries, nous avons constaté que le choix du traitement des ruptures récentes du tendon d'Achille ne fait l'objet d'aucun consensus et que toutes les modalités thérapeutiques sont possibles. Dans notre série, tous les patients ont bénéficié d'un traitement chirurgical à ciel ouvert utilisant différentes techniques: sutures simple avec surjet de renforcement ou laçage et plasties. Weber et al [6] et Farizon ont aussi privilégié la suture et laçage avec un éventuel renforcement s'il existe une fragilité tendineuse. Rouvillain [7], Boukhris [8] et beaucoup d'autres auteurs ont privilégié la ténorraphie percutanée selon la technique de Delponte alors que Lansdaal [9] a utilisé la chirurgie mini- invasive. Richard et al [10] ont utilisé le traitement fonctionnel Figure 3 et Figure 4.

Nous retrouvons dans notre étude 1.8% de complications mineures représentées par un cas d'infection superficielle du site opératoire. Nos résultats sont inférieurs aux travaux de Wong et al. [11] ou encore ceux de Strauss [12], à Beskin [13] qui retrouvent 7% des complications locales et nettement inférieurs à Khan et al. [14] avec 34%. Aucun cas d'atteinte du nerf sural n'a été rapporté. Il en est de même pour les séries de Delponte [15] et Kouvalchouk [16]. Aucun cas d'accidents trombo-embolique n'a été rapporté. Il en est de même pour les séries de Rettig [17] et Bruggeman [18]. Le retour à la vie active après chirurgie est un paramètre important. Le délai de reprise du travail dans notre étude était en moyenne de 3 mois. La reprise des activités sportives au niveau antérieure n'a été possible que pour 30% dans notre série, pourcentage nettement inférieur aux chiffres retrouvés dans la littérature: 78% pour Farizon après chirurgie conventionnelle, 55,5% pour Lecestre et 64,3% pour Rouvillain après ténorraphie percutanée. L'importance de l'amyotrophie est fonction de la durée d'immobilisation d'une part, et de l'efficacité de la rééducation fonctionnelle d'autre part. McComis [19] a observé que l'amyotrophie n’était pas un critère très fiable. Dans notre série, l'appui monopodal a été possible chez tous nos patients, ce qui est proche de ceux de Rouvillain et Farizon. Dans notre série, les amplitudes articulaires ont été approximativement les même < 5° par rapport au côté sain. Rouvillain a retrouvé un déficit de la flexion dorsale chez 6,7% de ses patients. Pour Farizon la mobilité a été symétrique au côté opposé sauf dans 2 cas (4,76%) où il existait une perte de la flexion dorsale de 10°. Lecestre a retrouvé une mobilité normale dans 96,72% des cas pour la flexion plantaire et dans 91,8% pour la flexion dorsale.

## Conclusion

Les ruptures du tendon d'Achille touchent préférentiellement le jeune sportif de sexe masculin. La qualité du résultat fonctionnel est capitale pour la reprise de l'activité sportive. La majorité des auteurs s'accorde à dire que La chirurgie à ciel ouvert avec mobilisation précoce est probablement la méthode de choix. Enfin, on constate d'après le suivi évolutif et la satisfaction des patients opérés au sein de notre service qu'il n'existe pas de supériorité entre la technique de Bosworth et celle de Chigot et qu'il s'agit beaucoup plus d'une préférence personnelle du chirurgien à telle technique ou l'autre.
